# Anti-cancer potential of casein and its derivatives: novel strategies for cancer treatment

**DOI:** 10.1007/s12032-024-02403-8

**Published:** 2024-07-11

**Authors:** Daniel Romero-Trejo, Itzen Aguiñiga-Sanchez, Edgar Ledesma-Martínez, Benny Weiss-Steider, Edith Sierra-Mondragón, Edelmiro Santiago-Osorio

**Affiliations:** 1 Hematopoiesis and Leukemia Laboratory, Research Unit on Cell Differentiation and Cancer, Faculty of High Studies Zaragoza, National Autonomous University of Mexico, 09230 Mexico City, Mexico; 2 Department of Biomedical Sciences, School of Medicine, Faculty of High Studies Zaragoza, National Autonomous University of Mexico, 56410 Mexico City, Mexico; 3 Department of Physiology, Biophysics, and Neurosciences, Center for Research and Advanced Studies of the National Polytechnic Institute, 07360 Mexico City, DF Mexico

**Keywords:** Milk protein, Casein, Cancer, Apoptosis, Signaling pathways

## Abstract

Cancer is one of the leading causes of death worldwide, with over 10 million fatalities annually. While tumors can be surgically removed and treated with chemotherapy, radiotherapy, immunotherapy, hormonal therapy, or combined therapies, current treatments often result in toxic side effects in normal tissue. Therefore, researchers are actively seeking ways to selectively eliminate cancerous cells, minimizing the toxic side effects in normal tissue. Caseins and its derivatives have shown promising anti-cancer potential, demonstrating antitumor and cytotoxic effects on cells from various tumor types without causing harm to normal cells. Collectively, these data reveals advancements in the study of caseins and their derivative peptides, particularly providing a comprehensive understanding of the molecular mechanism of action in cancer therapy. These mechanisms occur through various signaling pathways, including (i) the increase of interferon-associated STAT1 signaling, (ii) the suppression of stemness-related markers such as CD44, (iii) the attenuation of the STAT3/HIF1-α signaling, (iv) the down-expression of uPAR and PAI-1, (v) the loss of mitochondrial membrane potential and reduced intracellular ATP production, (vi) the increase of caspase-3 activity, and (vii) the suppression of TLR4/NF-кB signaling. Therefore, we conclude that casein could be an effective adjuvant for cancer treatment.

## Introduction

In 2020, the World Health Organization (WHO) reported 18.1 million cancer cases, and 9.6 million cancer deaths in 2018, with a projected increase to 29.4 million cases in 2040. The highest incidence among males was observed for lung cancer (14.3%), prostate cancer (14.1%), and colorectal cancer (10.6%). For females, breast cancer (24.5%), colorectal cancer (9.4%), and lung cancer (8.4%) were most frequently affected being a global health problem. In both sexes, lung, breast, and colorectal cancer are the leading causes of death worldwide by cancer [1]. Although tumors can be surgically removed and treated with chemotherapy, radiotherapy, immunotherapy, hormonal therapy, or combined therapies, current treatments often result in toxic side effects in normal tissue. Despite knowledge of the genetic basis and molecular mechanisms of this disease, cancer remains a highly aggressive pathology with a high mortality rate. This is primarily due to chemoresistance developed by tumor cells, metastasis formation, and the highly cytotoxic side effects in normal tissues [2–4]. Therefore, there is an urgent need to study new therapeutic molecules that can eradicate cancer cells while minimizing toxic side effects on healthy organs. In this regard, bioactive peptides obtained from milk show promise as antitumor agents, limiting the growth of cancer cells and, at the same time, positively influencing immune system activation [5–9]. Bovine milk has been a fundamental dietary for numerous human populations worldwide. National and international dietary guidelines recommend regular intake of milk and dairy products as part of a healthy diet [10]. The dairy group supplies many nutrients, including calcium, phosphorus, vitamin A, vitamin B12, vitamin D, riboflavin, proteins, essential amino acids, potassium, magnesium, selenium, and zinc [11].

Milk acts as the exclusive nutritional source until weaning, providing all the necessary components for development, including proteins, enzymes, carbohydrates, vitamins, and minerals, and ensuring a functional immune response [12]. Milk proteins can exert a wide range of positive effects on the body, such as boosting the immune system, protecting against harmful bacteria, viruses, and yeasts, and supporting the growth and proper functioning of the digestive system [13]. Interestingly, milk itself possesses tumor suppressor properties in many types of cancer [14–18]. This review provides a current summary of milk proteins, mainly caseins and peptides derived from casein, and explores their potential biomedical relevance in defending against the development of cancer.

## Milk composition

**Fig. 1:**
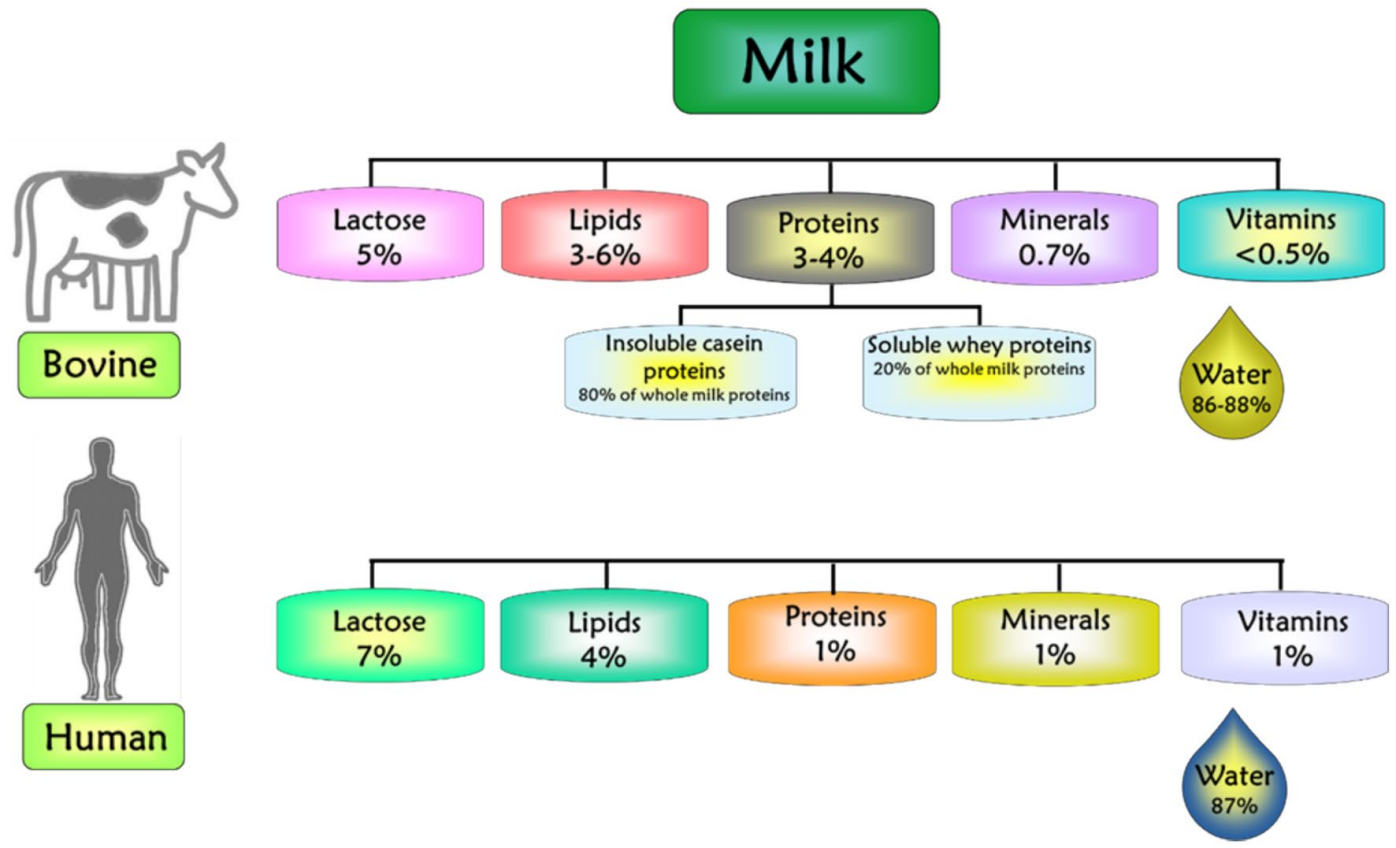
Approximate composition of bovine and human milk. Proteins are divided into insoluble casein proteins and soluble whey proteins

Bovine milk is an emulsion composed of proteins (3–4%), lipid (3–6%), carbohydrates (5%), minerals (0.7%), vitamins (0.5%), water (86–88%), and various additional elements, whereas human milk contains 1% protein, 4% lipid, 7% carbohydrates, 1% minerals (Calcium, Phosphorus, Magnesium, Potassium, Sodium), vitamins, and 87% water, as shown in Fig. 1. The composition of each one reflects the nutritional requirements for the growth and development of each species [19–22].

Bovine milk has been an essential dietary component for numerous human populations worldwide, constituting an almost universal element of human nutrition, regardless of consumer age [23–25]. In this context, bovine milk stands out as the most extensively studied among all mammals due to its high consumption and wide range of positive effects on the body. Thus, our emphasis is directed toward bovine milk, specifically the protein fraction.

**Fig. 2:**
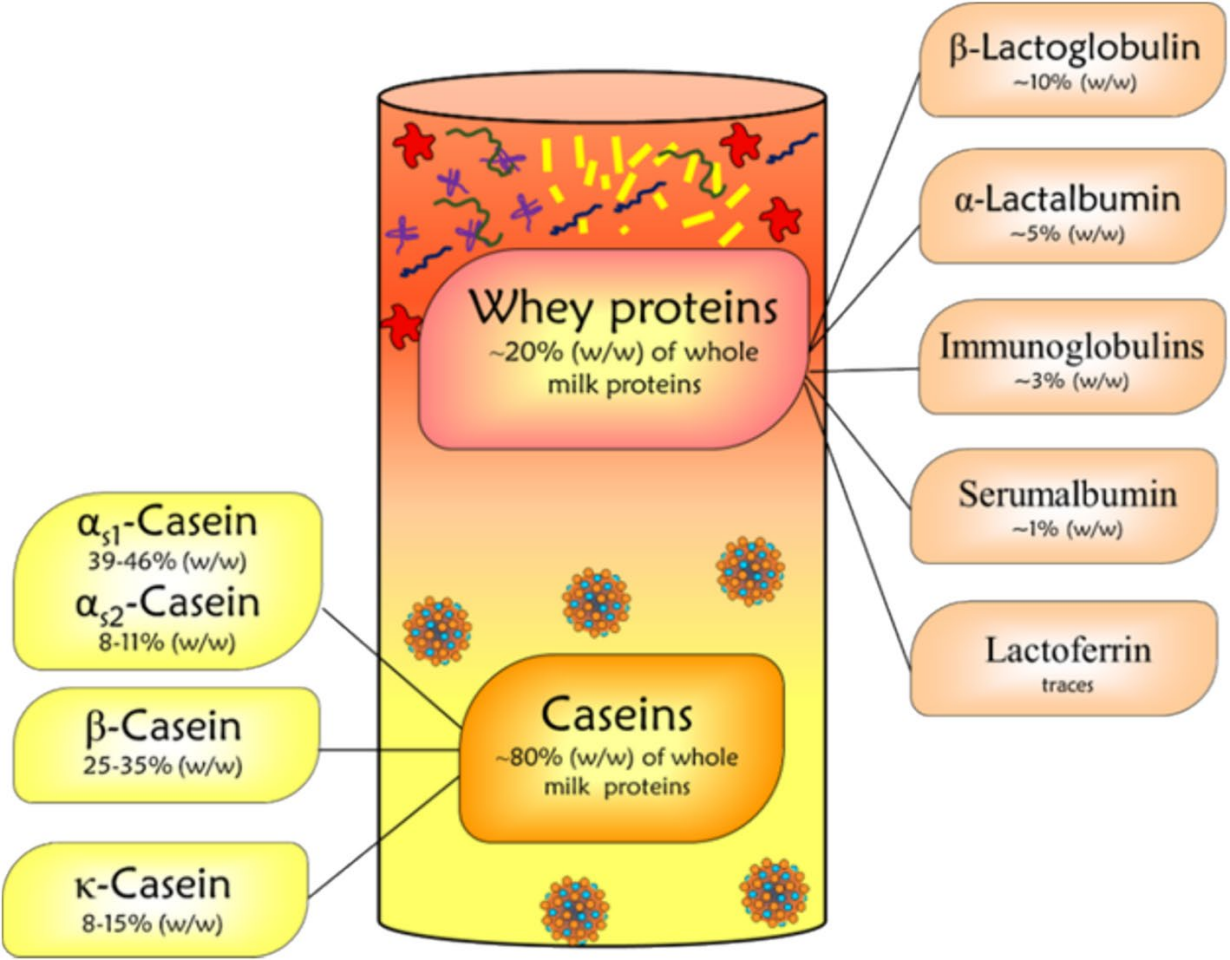
Overview of the caseins and whey proteins, and their share in the total protein fraction

Proteins in milk can be categorized into two main groups based on their solubility at pH 4.6 (isoelectric point of casein): (1) Caseins and (2) Whey proteins. Caseins are the most abundant proteins in milk, constituting approximately 78–80% of milk proteins with a milk content of 24–28 g/L [26–29]. The different casein fractions in bovine milk are diversified into four families based on the homology of their primary amino acid sequences, namely αs1 (39–46% of total casein), αs2 (8–11%), β (25–35%), κ (8–15%), and γ (3%) casein which is a natural degradation product of β-casein [22, 30–34]. In milk, caseins interact with calcium phosphate, forming large stable colloidal particles termed micelles with a size ranging from 30 to 300 nm. These micelles make possible the maintenance of a supersaturated concentration of calcium phosphate in milk, providing newborns with sufficient calcium phosphate for the mineralization of calcifying tissues [35]. On the other hand, the whey protein fraction constitutes < 20% of whole milk protein and is composed of α-lactalbumin (5%), β-lactoglobulin (10%), bovine serum albumin (1%), immunoglobulins (3%), and minor proteins such as lactoperoxidase, lysozyme, lactoferrin, and transferrin [22] as shown in Fig. 2. Whey proteins possess antiviral, bactericidal, antifungal, anti-inflammatory, and anti-oxidant properties [36–39].

## Caseins and casomorphins

**Fig. 3:**
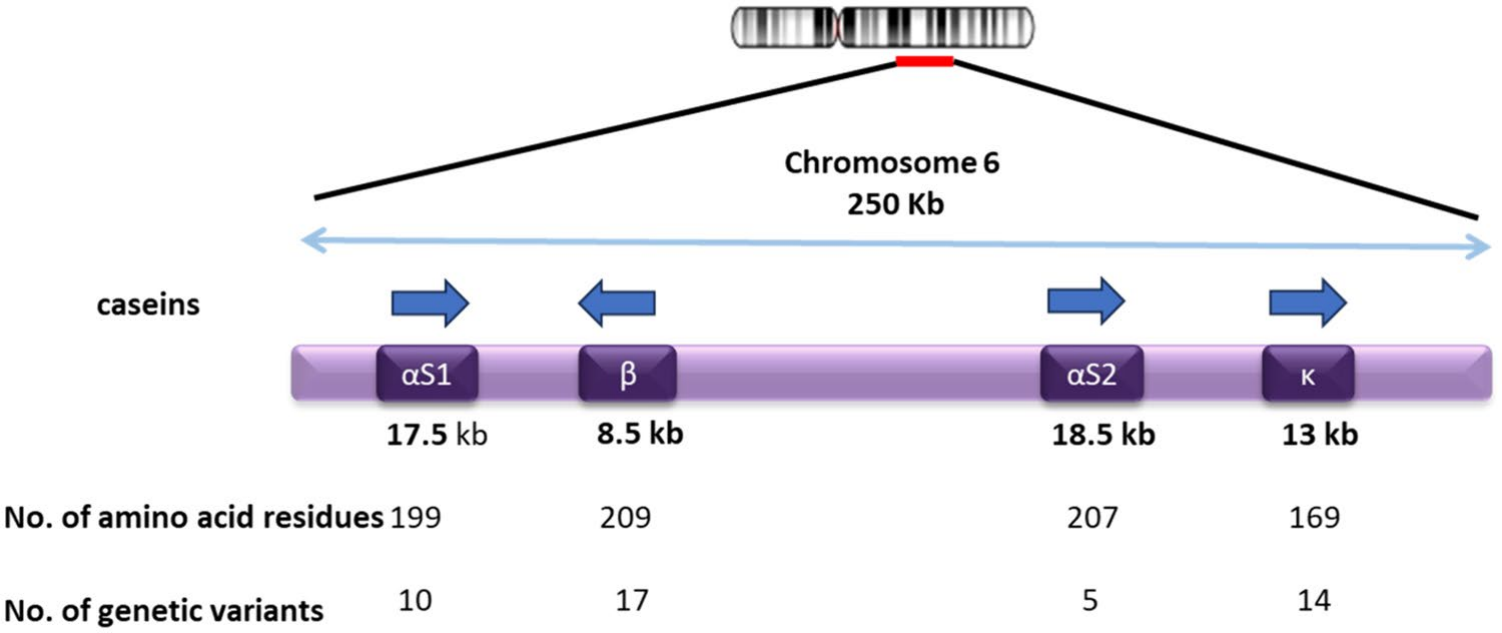
Genomic organization of the locus of bovine/goat αs1-, β-, αs2-, and κ-casein genes located on chromosome 6. The single arrow represents the direction of transcription of each gene. The double arrow indicates the distance in kilobases of casein genes. The number of amino acid residues and genetic variants from the corresponding genes are given under each gene

The caseins, main milk proteins, are encoded by a gene family covering a genomic region of 250 kb, located on chromosome 6 (6q31) in bovine cattle [34]. The genes CSN1S1, CSN2, CSN1S2, and CSN3 encode for αs1-, β-, αs2-, and κ-casein, respectively, as shown in Fig. 3. The first three genes are located in a locus that covers a region of 140 kb, whereas the κ-casein gene is located in a region of 95–120 kb.

Bovine caseins are synthetized and regulated in the mammary gland under hormonal control. All casein families have multiple genetic variants with different amino acid substitutions [19], resulting in a multitude of active protein fragments after hydrolysis during gastrointestinal digestion or food processing [25, 40, 41]. In milk, for example, β-casein is a protein encoded by the CSN2 gene, composed of 209 amino acids [42], and constitutes up to 37% of the caseins in bovine milk. The gene CSN2 is the most polymorphic among the genes encoding for caseins, with 13 genetic variants (A1, A2, A3, A4, B, C, D, E, F, G, H1, H2, I). The substitution of a single amino acid at the 67 position (histidine in A1 β-casein and proline in A2 β-casein) allows the distinction between two types of milk [43].

Several clinical trials have showed a relationship between the consumption of dairy products and a reduced risk of heart diseases. This association has been linked to the angiotensin-converting enzyme (ACE) inhibitory activity of whey and casein proteins, as well as their peptide derivatives released during digestion [44, 45]. Caseins and whey proteins have also been shown to exhibit anti-inflammatory properties, reducing T-cell activation markers in low-grade inflammation associated with obese human subjects [46]. In the context of antitumoral activity, in vitro studies have shown that three casein family members (αs1-, β-, and κ-casein) can all significantly inhibit the proliferation of human and mouse leukemic cells and murine mammary tumor cells in a concentration-dependent manner. Furthermore, Ramos et al. showed that sodium caseinate, a salt of casein, also inhibits the proliferation of mouse leukemic cells in vitro and in vivo [47, 48]. Additionally, different active protein fragments of β- and κ-casein have demonstrated antitumor effects against different types of tumors, including breast, melanoma, and ovarian cancer, which will be further studied later [22, 49–51].

On the other hand, casomorphins are a group of exogenous opioid-like peptides derived by the enzymatic action of α- and β-caseins. β-casomorphins are peptides of 4 to 11 amino acids encrypted in an inactive form and are released during digestion in vitro and in vivo. The first casomorphin isolated from an enzymatic casein digest was the β-casomorphin-7 following of β_b_-casomorphins-4, -5, and -6 [52], whereas opioid peptides isolated from α- and κ-casein digestion are named α-casomorphin (exorphins) and casoxins, respectively [53, 54]. In the context of antitumoral activity, various peptides derived from the digestion of casein have demonstrated antimutagenic properties against several types of cancer. Hatzoglou et al. reported that five different casomorphins (α-casein fragments 90–95 and 90–96, β-casomorphins-7 fragment 60–66, β-casomorphins-5 fragment 60–64, and the morphiceptin, the amide of β-casomorphins-4) have anti-proliferative activity on T47D breast cancer cells. These peptides increase the number of cells in G0/G1 phase and significantly diminish the percentage of cells at S and G2/M phases, indicating cell cycle inhibition [55].

Additional findings indicated a dose-dependent reduction in cell proliferation by various casomorphin peptides on human prostatic cancer cell lines (PC-3, DU 145, and LNCaP), through partial interaction with opioid receptor binding sites [56]. Furthermore, β-casomorphins-7 and the phosphopeptide β-casein induced apoptosis in HL-60 leukemia cells [57]. The interaction between casomorphins and tumor cells takes place via δ- and κ-opioid receptors, with exception of morphiceptin, whose action is mediated by type II somatostatin receptor. In this case, the casomorphins exhibited different receptor affinities [22].

## Casein against breast cancer

Breast cancer represents the first incidence and cause of death in woman worldwide [58]. Risk factors such as early menarche, late first pregnancy (after 30), and late menopause are all associated with an increase in developing breast cancer [59–61]. In contrast, early first pregnancy, multiple pregnancies, and extended periods of lactation reduce risk of developing breast cancer [62]. Sotgia et al. showed that the implantation of mammary tumor cells within the mammary gland of a constitutively lactating mouse model (Cav-3 (-/-) mice) inhibited tumor growth by over 1000-fold. Furthermore, in vitro studies show that the addition of human milk at low concentrations to cultured mammary tumor cells reduces their capacity for migration [63]. There are several potential explanations for why extended lactation might offer a protective effect. For example, it may limit the exposure of the breast to the inflammatory environment of involution or prevent potentially transformation from normal to pre-cancerous cells, as well as the progression from pre-cancerous to tumor cell [64]. These results suggest that milk has a protective effect against breast cancer, benefiting both the mother and infants by providing a source of macro- and micronutrients during breastfeeding. It confers protection against infections and childhood cancer [65, 66]. Furthermore, exclusive breastfeeding provides more beneficial immunological effects compared with that supplemented by alternative feeding [66].

One novel hypothesis against breast cancer is that milk itself contains, as yet, undiscovered components that function as tumor suppressors. Bonuccelli et al. showed that three members of the casein gene family (α-, β-, and κ-casein) can all significantly reduce the migration of murine mammary tumor cells (Met-1), as well as two human breast cancer cells (MCF10 and MDA-MB-231 cells), with α-casein being the most effective. Furthermore, recombinant expression of α-casein in mammary tumor cells remarkably attenuates both in vivo tumor growth (> fivefold) and experimental lung metastasis (> ninefold) in athymic nude mice by reducing the “stemness” and conferring a more “differentiated” mammary cell phenotype. This increases their sensitivity to apoptosis by STAT1 signaling [48]. In direct support of this notion, Garner et al. assessed the effect of α-casein on Breast Cancer Stem Cell (BCSC) activity in vitro and found that α-casein to significantly reduce BCSC in the triple-negative MDA-MB-231 cell line. This reduction is mediated by HIF-1alpha, a hypoxia-inducible transcription factor closely associated with the induction and maintenance of a BCSC phenotype [67–69], which reduces the “stemness” and confers more differentiated phenotype, increasing their sensitivity to apoptosis [48]. On the other hand, Richter et al. showed that recombinant expression of a fragment of human κ-casein (RL2) induces loss of mitochondrial membrane potential and reduces ATP levels in MCF-7 and MDA-MB-231 breast cancer cells, leading to cell death. Interestingly, there is strong evidence that fragment of human κ-casein specifically affects cancer cells and does not exert any suppressive action on normal cells or non-malignant mesenchymal stem cells [70]. In this regard, casein or peptides derived from casein are promising antitumor therapeutics for the treatment of breast cancer minimizing toxic side effects in healthy organs.

### Casein against melanoma

Melanoma is a highly metastatic malignant neoplasm that shows limited responsiveness to systemic treatments in patients with advanced stage. In 2020, there were 324,635 new cases of this disease, resulting in 57,043 deaths worldwide. Although new systemic treatments have been used for patients in stages III and IV, only 20% of patients have an effective response with maintenance of long‐term survival [71]. Thus, the high cost of treatment and the low effectiveness of available therapies highlight the urgent need for development of new therapeutic strategies.

The use of natural products in cancer therapy is an active area of research, and few studies have been conducted to evaluate the anti-cancer effects of milk proteins in melanoma. In direct support of this notion, Alexandre et al. demonstrated the antineoplastic effect of INKKI, a cationic peptide isolated from hydrolysis of bovine β-casein, in melanoma. They showed that INKKI peptide inhibits cell proliferation and has cytotoxic and apoptotic effects on B16F10 cells in vitro. Additionally, a 72.62% inhibition in tumor growth and a decreased number of metastases were observed in tumor-bearing mice treated with INKKI [51]. However, the exact cell-specific receptor and signal transduction pathways involved in INKKI’s action have not been fully investigated yet and require further research. Although evidence from the literature has indicated that β-casein protein and its derivate peptides are interesting compounds of milk with antineoplastic effects, few studies have attempted to understand their effects on melanoma. These findings suggest that components of milk as β-casein could be promising candidates for the development of new therapeutic agents against melanoma. However, stronger scientific evidence is needed to validate the effect of β-casein on melanoma cancer.

### Casein against ovarian cancer

Ovarian cancer is one of the most lethal gynecological malignancies, posing a significant threat to women’s health worldwide. Although ovarian cancer can be removed by surgical resection and treated with chemotherapy, problems continue to arise particularly with respect to chemotherapy due to side effects, drug resistance, and low specificity of currently available drugs [72]. Therefore, new therapeutic strategies need to be employed as anti-cancer agents to minimizing toxic side effects in healthy organs. Milk casein protein has been reported to have suppressor tumor activity toward other cancer types such as acute myeloid leukemia, melanoma, and breast cancer. However, little is known about the effect of milk casein on ovarian cancer. Wang et al. reported that PGPIPN, a hexapeptide derived from bovine β-casein, inhibited the proliferation of SKOV3 human ovarian cancer cell line, as well as primary ovarian cancer cells, in vitro. Consistently, they demonstrated that PGPIPIN also inhibits the primary tumor growth rate in xenograft ovarian cancer model mice in a dose-dependent manner by promoting cell apoptosis through inhibition of BCL2 pathway and caspase-3 activation. Interestingly, they also discovered that peptide derived from β-casein protein had no effects on the inhibition of proliferation in the human normal hepatic cell line LO2 and murine embryo fibroblast cells (MEFs), as compared with the traditional anti-cancer drugs (5-FU) [50]. These results are consistent with those reported in the literature [47, 73, 74], demonstrating that casein or peptides derived from casein do not have toxic effect on the proliferation of normal cells. This suggests that casein might be a potent therapeutic agent for the treatment of other types of cancer, such as ovarian cancer. However, more studies must be conducted for its use in ovarian cancer therapy.

### Casein against leukemia

Acute myeloid leukemia (AML) is a heterogeneous and aggressive form of blood cancer characterized by the uncontrolled proliferation of myeloid hematopoietic cells (myeloblasts) in the bone marrow, the spongy tissue inside bones where blood cells are made. It can quickly infiltrate in blood and tissues such as the spleen, liver, gums, and central nervous system [75] leading to the formation of metastases, the main cause of death by cancer [76]. The number of new cases among men and women per year is about 4.2 per 100,000 population, with an incidence of over 20,000 cases per year in the United States. Generally, older adults (> 80 years) are more likely to develop AML than younger adults or children. Approximately, 15–20% of pediatric acute leukemia cases and 80% of acute leukemia cases in adults are AML cases [75, 77–79].

For decades, the conventional treatment of AML has involved initial induction therapy and post-remission therapy. However, induction therapy is highly toxic to bone marrow, leading to pancytopenia, bleeding complications, gastrointestinal system issues, kidney failure due to tumor lysis syndrome, and electrolyte disturbances [75]. Although the goal of post-remission therapy is to prevent disease relapse by employing highly cytotoxic chemotherapies (such as high-dose cytarabine) or allogenic hematopoietic stem cell transplantation, many tumor cells can develop drug resistance during or after treatment, causing toxic side effects in normal hematopoietic cells.

Nowadays, the use of natural products in cancer therapy is an active area of research, and several studies have been conducted to evaluate the anti-cancer effects of leukemia in vitro and in vivo using milk proteins [80–83], which contain as-yet-undiscovered components that might function as tumor suppressors. In support of this idea, Ramos-Mandujando et al. showed that three casein family members (α-, β-, and κ-casein) can all significantly inhibit the proliferation of different leukemic cell lines (WEHI-3, J774, P388 and 32D cl3), with α-casein being the only one able to induce the differentiation of 32D cl3 (no malignant cells) into the monocyte-macrophage linage. They have also demonstrated that sodium caseinate (a salt of casein), the main milk protein, inhibits the proliferation of these leukemic cells in vitro [47] and leads to increased survival in vivo in J774 tumor-bearing mice [81]. Furthermore, Ledesma et al. reported that sodium caseinate inhibits the proliferation of tumor cells and enhances apoptosis in WEHI-3 cells through DNA fragmentation. Additionally, it promotes the proliferation of mononuclear normal cells from BALB/c mice bone marrow. Interestingly, they also showed that casein prolonged the survival of WEHI-3 tumor-bearing mice for more than 40 days, suggesting that this molecule is capable of reducing tumor growth of WEHI-3 cells in vivo [73].

The combined therapy has been considered to enhance its toxic effect toward cancer cells, reducing drug resistance and treatment duration compared to monotherapy [9, 83]. In this context, Aguiñiga et al. showed that combination of the IC25 of sodium caseinate-cytarabine or sodium caseinate-daunorubicin enhances the activity of the treatments achieving a 70% inhibition and death rate in WEHI-3 leukemic cells through activation of caspase 3. Additionally, combined therapy prolonged the survival of WEHI-3 tumor-bearing mice for more than 70 days compared to individual treatments. Interestingly, the authors also reported that the combination of sodium caseinate-cytarabine or sodium caseinate-daunorubicin enhances the proliferation of mononuclear normal cells from BALB/c mice bone marrow compared with control groups [74]. Theses result are consistent with those reported in the literature [47, 50, 73] demonstrating that casein or peptides derived from casein have an inhibitory effect on the proliferation of tumor cells without damage normal cells. These findings are very relevant and promising because several conventional therapies generate cytotoxic effects in both tumor and normal cells, harming normal tissues. However, further scientific studies should explore the effect of casein in other types of leukemias.

## Potential mechanism of action of caseins and its derivatives on tumor cells

**Fig. 4:**
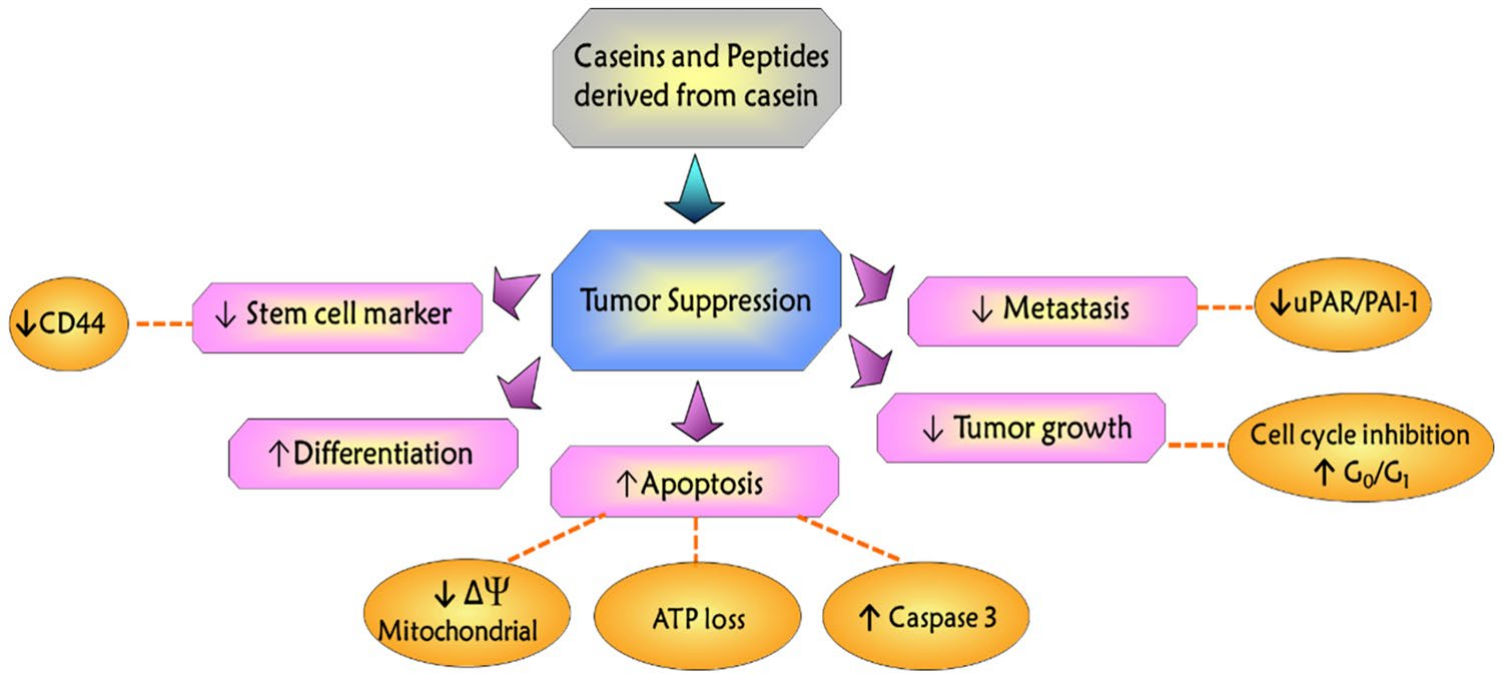
Mechanism of action of the casein and its derivatives on tumor suppression, differentiation, apoptosis, tumor growth, and metastasis in tumor cells

Casein, the major protein found in milk, and its derived peptides have been demonstrated to possess numerous therapeutic effects in several experimental models of cancer diseases [73, 74, 84–86]. A schematic summarized of the antitumor effects of caseins and their peptide derivatives is shown in Fig. 4. Previous studies have suggested that casein treatment might attenuate tumor progression. This beneficial effect occurs through (i) the suppression of stem cell markers (CD44) [48, 87] and cell adhesion molecules such as uPAR/PAI-1, which plays an important role in tumor progression and metastasis [88], (ii) the cell cycle inhibition [89], and (iii) the increase of cell death by apoptosis [70, 73, 74, 90, 91] and the anti-proliferative effect on tumor cells is partly attributed to the promotion of cell differentiation [48]. Therefore, the exploration of the antitumoral activity exhibited by caseins and their derivatives has prompted several experimental and theoretical studies to further understand its molecular mechanism of action.

Additionally, Bonuccelli et al. reported that the treatment with α-casein on murine mammary tumor cell lines and human breast cancer cell lines induced an increase of interferon-associated STAT1 signaling [48]. In general, STAT1 is recognized as a tumor suppressor. It is established that the downregulation of STAT1 activation and the reduction in its expression are prevalent in various tumoral cells [92, 93]. Additionally, a correlation of STAT1 expression in cancer patients tends to have a better clinical prognosis, including colon-rectal cancer, soft tissue sarcoma, metastatic melanoma, hepatocellular carcinoma, and pancreatic cancer [94–97]. Interestingly, the caseins (α, β, and κ) share a similar number of amino acid as the interferons (α, β, and γ). This suggests that caseins might also function as cytokines and play a role in the same molecular signaling cascade [48].

In canonical mechanisms of STAT1, its protein generally exists as an inactive form in the cytoplasm; STAT1 is initially phosphorylated and activated by the receptor-activated kinases such as JAK, in response to IFN stimulation. The active form of STAT1 then translocates to the nucleus, where it acts as a transcription factor regulating various aspects of tumor suppression, including cell grown arrest, apoptosis, and inhibition of angiogenesis [98, 99]. Thus, it is possible that casein family members could be used as novel biological molecules for cancer treatment by the activation of interferon signaling via upregulation and hyperactivation of STAT1. Furthermore, Wang et al. [100] demonstrated for first time that overexpression of STAT1 results in the suppression of stemness-related markers such as CD44, CD133, NANOG, and OCT4 in chemoresistant epithelial ovarian cancer cells and decreases tumorigenesis capacity. Importantly, α-casein overexpression in the triple-negative MDA-MB-231 breast cancer cell line resulted in the reduction of proportion of CD44 + cells [48]. CD44 is a cell surface marker, which is overexpressed on cancer stem cells, because it is an extracellular matrix adhesion protein. It plays a role in metastasis, cell adhesion, and migration [87, 101].

On the other hand, STAT3 plays an important role in tumor development and aberrant phosphorylation of STAT3 accumulates in nearly 70% of cancers and is associated with disease progression and poor prognosis. STAT3 acts as an oncogene, regulating various fundamental cellular processes, including proliferation, differentiation, angiogenesis, invasion, and metastasis. It can be activated by multiple proinflammatory factors and growth factors [102–104]. The inhibiting STAT3 activity is considered a viable strategy for cancer treatment [105–107]. Additionally, STAT3 can induce the expression of two prominent transcriptional targets, such as hypoxia-inducible factor-1α (HIF-1α) and vascular endothelial growth factor (VEGF) [108]. It has been reported that the STAT3/HIF-1α pathway is closely associated with the progression of various tumors, including prostate cancer, hepatocarcinoma, breast cancer, and ovarian cancer [109–111]. Garner et al. have reported that α-casein inhibits HIF-1α signaling in breast cancer cells. Moreover, α-casein conditioned media reduces STAT3 reporter activity, indicating that STAT3 is a crucial transcription factor in regulating HIF-1α in breast cancer stem cells [64]. Therefore, these data may be associated with the mechanisms by which α-casein reduces stem cell activity in vitro, and STAT3 was identified as a regulator of pro-tumorigenic HIF-1α signaling.

Previous studies have reported that members of the cell-associated fibrinolytic system (urokinase plasminogen activator and its receptor, uPA/uPAR) as well as plasminogen activator inhibitor type-1 (PAI-1) act as prognostic/predictive biomarkers of malignancy [112–114]. The literature has reported that HIF-1α activation promotes the transcriptional activation of uPAR in cancer cells [115–117]. On the contrary, it has been described in triple-negative MDA-MB-231 breast cancer cells that the overexpression of casein induces a low or undetectable uPAR and PAI-1 protein expression [48].

Caseins and their peptides derived from caseins are multi-functional, exerting effects such as anti-microbial, immunomodulatory, anti-oxidant, anti-metastasis, and apoptotic. Azevedo et al. reported that the peptide INKKI, corresponding to bovine β-casein residues 41–45, induced apoptosis in B16F10 cells in a caspase-dependent manner by increasing caspase-3 activity and suggested that this process occurs through the mitochondrial pathway [51]. Additionally, INKKI significantly reduced tumor growth in a model of melanoma. Likewise, MCF-7 human breast adenocarcinoma cells treated with the peptide INKK showed cell arrest in the G0/G1 phase and decreased expression of cyclin D1 [118]. On the other hand, PGPIPN, a hexapeptide derivate from bovine β-casein residues 63–68, inhibited the proliferation of human ovarian cancer cells as well as the primary tumor growth via downregulation of BCL-2 signaling [50].

The recombinant Lactaptin 2 (RL2) is comprised of the amino acids 23–134 of human κ-casein. RL2 was shown to induce cell death in MDA-MB-231 and MCF-7 breast cancer cells, suppress tumor growth, and metastasis in mice [119, 120]. Richter et al. reported that the interaction of RL2 with the TOM70 protein induces a loss of mitochondrial membrane potential, downregulates intracellular ATP production, reduces cell viability, and increases cell death in breast cancer cells [70]. Wohlfromm et al. have described that RL2 peptide enhances cell death in combinatorial treatments with drugs such as doxorubicin, inducing the intrinsic apoptosis pathway in triple-negative breast cancer cells [90]. Therefore, this evidence suggests that caseins and their derivatives are considerably antitumor candidates.

On the other hand, several studies have shown that the inflammation could play a dual role in cancer. Some studies suggest that chronic inflammation in tumor cells promotes the release of cytokines leading to an inflammatory microenvironment and facilitating the occurrence and progression of tumors. However, other reports described that the induction of acute inflammation is a potential strategy for the treatment of cancer [121]. The Toll-like Receptor 4 (TLR4) signaling pathway results in the activation of nuclear factor-kappa B (NF-κB) and the subsequent initiation of the inflammatory responses [122–124]. Recently, TLR4 has been described to be highly expressed in diverse types of cancer, including colon cancer, hepatocarcinoma, ovarian cancer, lung cancer, and breast cancer [122, 125, 126], promoting inflammation, tumor growth, invasion, and metastasis of cancer cells [127]. However, Ahmed et al. reported that silencing of TLR4 promote tumor progression and metastasis in murine model of breast cancer [128]. In this context, it has been documented that human αs1-casein activates the secretion of proinflammatory cytokines, such as GM-CSF (granulocyte macrophage colony-stimulating factor), IL1-β (interleukin 1β), IL-6 (interleukin 6), and chemokine IL-8 (interleukin 8) in human monocytes TLR4 signaling pathway [129, 130]. On the other hand, Liu et al. have reported that β-casein and its peptide QEPVL have an anti-inflammatory effect and attenuated inflammation through NF-κB/NLRP3 signaling pathway in mice with ulcerative colitis [131]. Therefore, it is possible that the caseins and its derivatives have the ability to regulate inflammatory response in cancer cells and the tumoral microenvironment.

**Fig. 5:**
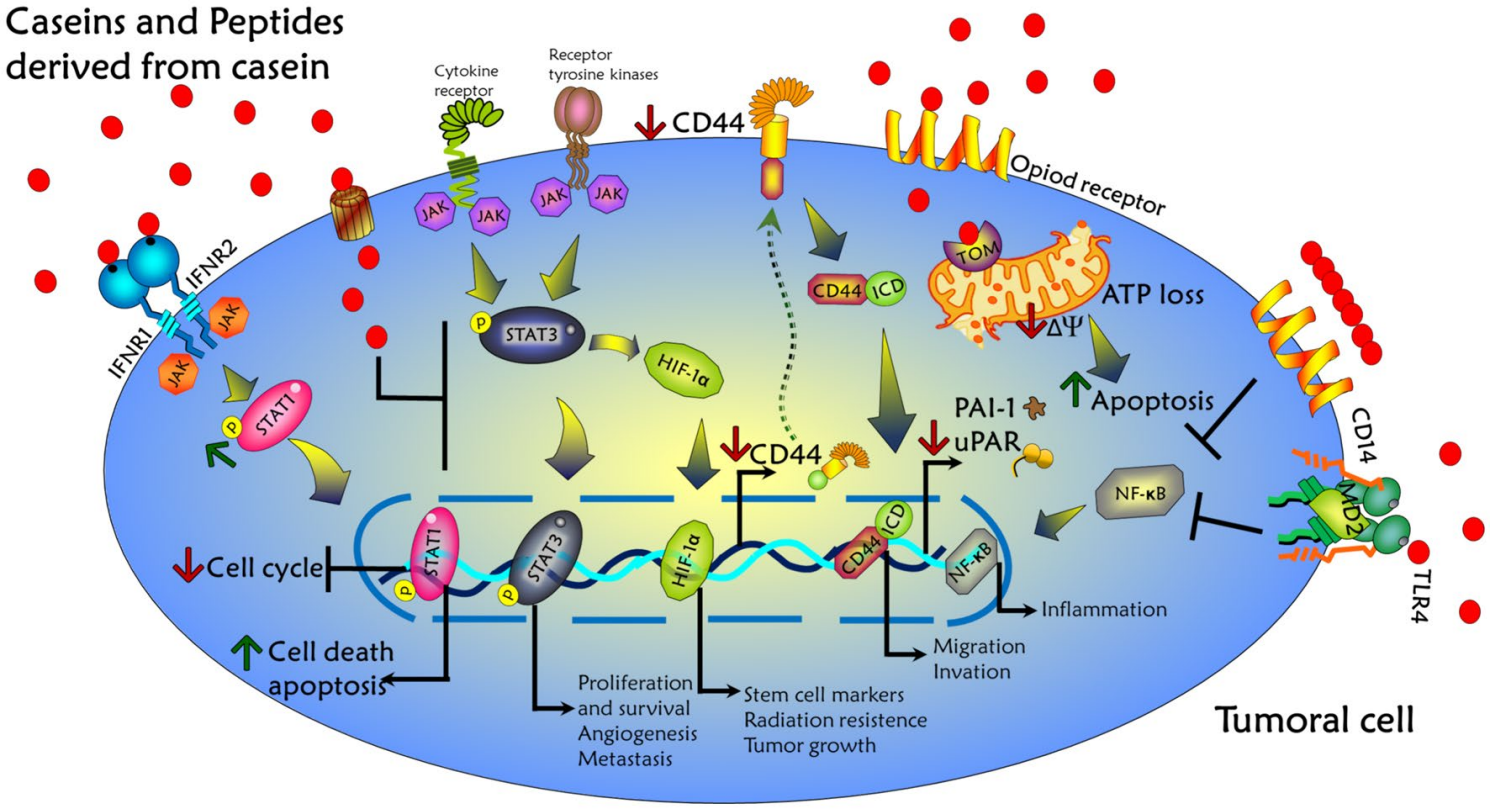
Signaling pathways by which caseins induce biological activities in tumor cells. Caseins and peptides derived from casein bind to several cell surface binding sites to activate the JAK/STAT1 pathway and induce the arrest of cell cycle and apoptosis. On the other hands, peptides derived from casein also downregulate JAK/STAT3, HIF1α, CD44, and NF-κB activation; it reduces cell proliferation, angiogenesis, tumor growth, inflammation, metastasis, and the mitochondrial membrane potential inducing ATP loss and apoptosis in cancer cells

This review focuses on advances in the study of caseins and their peptides and particularly provides a comprehensive understanding of molecular mechanism of action in cancer therapy. These mechanisms occur through various signaling pathways, including (i) the increase of interferon-associated STAT1 signaling, (ii) the suppression of stemness-related markers such as CD44, (iii) the attenuation of the STAT3/HIF1-α signaling, (iv) the down-expression of uPAR and PAI-1, (v) the loss of mitochondrial membrane potential and reduced intracellular ATP production, (vi) the increase of caspase-3 activity, and (vii) the suppression of TLR4/NF-кB signaling. An integrative scheme of these mechanisms is shown in Fig. 5. Therefore, the information presented in this paper identifies caseins and its derivatives as a possible potential therapeutic agent for cancer treatments.

## Conclusions

Diverse research highlights the antitumoral potential of casein and its derivatives in preclinical trials. Their therapeutic effects are thought to be partly mediated through the regulation of cell signaling pathways, as summarized in this article. This includes increase in STAT1 signaling, the promotion of apoptosis (caspase-3 activity and loss of mitochondrial function), the suppression of STAT3, HIF-1α, TLR4/NF-κB signaling, and the downregulation of CD44, uPAR, and PAI-1. These actions result in the modulation of key cancer hallmarks such as cell proliferation, angiogenesis, migration, stem cell markers, apoptosis, inflammation, and metastasis.

Future research will delve deeper into the signaling pathways mediated by caseins and their peptides, thereby elucidating the comprehensive mechanism of action of these compounds across various types of cancer. Also, further research is needed to determine the optimal dose, bioavailability, and bioefficacy of caseins or their peptides. However, clinical studies are required to fully understand the effects of caseins or its peptides in humans. The future perspectives of caseins and its peptides in cancer and human health revolve around advancing its precision through personalized medicine, exploring synergies with other treatments, understanding resistance mechanisms, and conducting rigorous clinical research for evidence-based integration into medical practice.

## Data Availability

No datasets were generated or analysed during the current study.
